# Effect of medium-chain triglycerides supplements and walking on health-related quality of life in sedentary, healthy middle-aged, and older adults with low BMIs: a randomized, double-blind, placebo-controlled, parallel-group trial

**DOI:** 10.3389/fnut.2023.1296896

**Published:** 2023-11-23

**Authors:** Haruna Ishikawa, Keiichi Kojima, Shinji Watanabe, Naohisa Nosaka, Tatsushi Mutoh

**Affiliations:** ^1^Central Research Laboratory, The Nisshin OilliO Group, Ltd., Yokohama, Japan; ^2^Department of Aging Research and Geriatric Medicine, Institute of Development, Aging and Cancer, Tohoku University, Sendai, Japan; ^3^Research Institute for Brain and Blood Vessels, Akita Cerebrospinal and Cardiovascular Center, Akita, Japan

**Keywords:** medium-chain fatty acids, medium-chain triglycerides, octanoic acid, decanoic acid, tiredness, vitality, mental health, subjective fatigue

## Abstract

**Introduction:**

To extend individuals’ healthy life expectancies, the improvement of subjective health and quality of life (QOL) has been increasingly prioritized, alongside the improvement of their physical functioning. Reports have indicated that intake of medium-chain triglycerides (MCTs) benefits the physical health of older individuals requiring nursing care, and athletes, and healthy individuals. But there are few studies investigating the effects of MCTs on subjective health and QOL. The present study sought to evaluate the combined effects of 12-week MCTs supplements and moderate-intensity walking exercise on the subjective health and QOL of middle-aged and older adults aged 60–74 with low BMIs (< 24 kg/m^2^) and who had no exercise habits.

**Methods:**

A placebo-controlled, double-blind, parallel-group trial was conducted. Three MCTs supplement groups with different doses and fatty acid compositions were compared with a control group. The study used the SF-36v2 questionnaire to assess subjective health and health-related QOL (HRQOL).

**Results:**

The result showed significant improvements in the scores on subscales of the physical QOL, such as Physical functioning and General health, and summary scores on the mental QOL, compared to the control.

**Conclusion:**

It is estimated that the combination of continuous intake of MCTs and walking exercise may affect HRQOL and improve subjective physical and mental health in sedentary, healthy, middle-aged and older adults.

**Clinical trial registration:**

https://rctportal.niph.go.jp/s/detail/um?trial_id=UMIN000046861, UMIN000046861.

## 1 Introduction

In Japan in 2021, the percentage of people aged 65 years and older comprised 28.9% of the total population. This is one of the highest such percentages in the world, and it is estimated to reach 31.2% by 2030 (1). Among this aging population, the number of people requiring long-term care and support continues to increase (2, 3). This trend not only leads to a decrease in their quality of life (QOL) (4), but also contributes to the costs of health care and long-term care (5, 6). Furthermore, the gap between average life expectancy and healthy life expectancy (i.e., the period during which some kind of support or long-term care is required) has remained at around 10 years for both men and women over the past dozen years (1). Increasing the number of people who can live independently, and extending healthy life expectancy, also reduces the burden on the people around them.

In the prevention of the necessity of long-term care, efforts have been made to improve not only the physical functions of older people, but also their QOL and subjective health status (7). It has been reported that improving physical function alone does not improve QOL, including subjective health status, in community-dwelling older adults in Japan (8). Additionally, subjective health has been reported to correlate with healthy life expectancy (9). Therefore, it is important to address not only the physical aspects of health, but also to improve QOL and subjective health in order to extend healthy life expectancy.

Medium-chain fatty acid (MCFA) is the broad term used to refer to straight-chain saturated fatty acids with 6–12 carbon chains. Medium-chain triglycerides (MCTs) composed of MCFA have the property of being preferentially converted into energy in the body after ingestion, unlike ordinary fats and oils, which consist of long-chain triglycerides (LCTs) (10). In clinical studies, a continuous daily consumption of 6 g of MCTs has been reported to improve physical function and body composition in older people. At a long-term care hospital, this MCTs intake led to an improvement in blood albumin levels, an indicator of malnutrition (11). In addition, significant improvements in grip strength, walking speed, and muscle mass were observed in nursing home residents (12, 13). Furthermore, animal studies have reported that continuous intake of MCTs increased mitochondrial biosynthesis (14) and activation of pathways involved in albumin synthesis in the liver (15). A case study of athletes reported a reduction in subjective fatigue with continuous intake of MCTs (16).

Regarding the effects of MCTs on mitochondrial function, which is associated with fatigue, animal studies have reported that both octanoic acid and decanoic acid increase mitochondrial biosynthesis and fatty acid oxidation-related gene expression, respectively (17, 18). Continuous intake of MCTs has been reported to increase fat oxidation during exercise in normal weight (19) and overweight (20) individuals having no exercise habits. Thus, continuous consumption of MCTs may benefit the physical health of people who need long-term care, and athletes, and normal-weight and overweight individuals. However, few studies have examined the effects of MCTs supplements on QOL and subjective health, and the effects of differing MCFA compositions and intake of MCTs are unknown.

In this study we investigated the effects of continuous intake of MCTs and moderate-intensity exercise on QOL and subjective health in healthy, middle-aged and older men and women with low BMIs, who had no exercise habits. In addition, three MCTs supplement groups were established to investigate the effect of MCTs doses and fatty acid composition.

## 2 Methods

Details of ethics, sample size, inclusion and exclusion criteria of subjects, study design and allocation of participants, management during the intervention period, dietary intervention, physical exercise intervention, lifestyle survey, dietary survey were described previously (21).

### 2.1 Ethics

The study was conducted in compliance with the Declaration of Helsinki (revised in 2013), the Japanese Ethical Guidelines for Medical and Health Research Involving Human Subjects, and the Japanese Act on the Protection of Personal Information (22, 23). We obtained approval from the ethics committees of the Yoga Allergy Clinic (approval number 21000023, approval date May 13, 2022). We registered this clinical trial in UMIN-CTR before the recruitment of participants (UMIN000046861).^1^ The measurement of HRQOL was a secondary endpoint of this study.

### 2.2 Sample size

In a previous study of MCTs in a test diet, their effect on physical function was confirmed in 21 cases; older adults in nursing homes, with BMIs of 23 kg/m2 or less and aged 65 years or older, were fed 6 g of MCTs per day for three months (12). In the present study the amounts of MCTs in the test supplement were 6 g and 2 g, being equal to and one-third of the amount in the previous study. The number of cases in this study was set at 30 per group (120 in total), on the assumption that the number of cases required would increase and on the expectation that there would be drop-outs during the study and that some patients would be ineligible for analysis.

### 2.3 Subjects

Participants were Japanese males and females aged 60–74 years, with a BMI in the range from 19 kg/m^2^ to less than 24 kg/m^2^, who exercise or walk less than once a week, and for less than 30 min each time, and who did not routinely (for more than four hours a week) provide childcare or nursing care. Individuals who were instructed by a doctor to refrain from walking or exercising, or who were serious illness were excluded.

### 2.4 Study design and allocation of participants

The study was conducted as a randomized, double-blind, parallel-group trial with a placebo as the control diet. A party not involved in the trial (the test diet allocation manager) divided the subjects into four groups by stratified randomization: test diet groups (three types) and control diet group. The allocation factors were age, sex, and blood albumin level. After allocation, the test diet allocation manager confirmed that there were no significant differences between the four groups in age, sex, and blood albumin levels. The test diet allocation manager kept the test diet allocation list strictly confidential until the key was opened, and blinding was maintained for all parties except the test diet allocation manager.

### 2.5 Management during the intervention period

During the study period, the participants were instructed to avoid excessive physical activity, other than exercise interventions, to maintain normal lifestyle habits. If they performed non-routine household chores requiring physical effort (cleaning, redecoration and repair of the house, shoveling snow) or recreational trips for unavoidable reasons, they were instructed to record the details of these activities on the lifestyle questionnaire.

### 2.6 Dietary intervention

During the study period, one packet of the supplement containing 3 g of oil was taken as a supplement after any two of the three daily meals, breakfast, lunch, and dinner (two packets per day, 6 g of fats and oils). The oils in the supplements were LCTs (rapeseed oil from The Nisshin OilliO Group, Ltd., Tokyo, Japan) and MCTs (Nisshin MCT oil, Nisshin MCT-C10R, also from The Nisshin OilliO Group, Ltd.).

The four supplements were: the control supplement, Decanoic acid supplement, High-dose octanoic acid supplement, and Low-dose octanoic acid supplement. The daily amounts of LCTs, MCTs, and MCFAs contained in MCTs per 6 g of oil in the supplements are shown in Table 1. The subjects were instructed to maintain their pre-intervention dietary habits during the study period, except for the consumption of supplements.

**Table 1:** Content of LCTs, MCTs, and MCFAs of MCTs in 6 g of oil in the supplement.

		Control supplement	Decanoic acid supplement	High-dose octanoic acid supplement	High-dose octanoic acid supplement
LCTs	g/day^1^	6	-	-	4
MCTs	g/day^1^	-	6	6	2
Octanoic acid (8:0)	g/day^2^	(−)	(1.40, 1.95)	(3.72, 4.14)	(1.24, 1.38)
Decanoic acid (10:0)	g/day^2^	(−)	(3.47, 4.00)	(1.06, 1.46)	(0.352, 0.487)

^1^Values show the amount of content. ^2^Lower and upper limits are shown. -, (−) Content is zero.

The three MCT supplement groups were established for the following reasons. First, the MCTs (High-dose octanoic acid supplement in this study) which Abe et al. found to improve muscle strength and other parameters in persons requiring nursing care, were established to establish whether there would be a similar improvement in the subjects in this study (13). Second, a reduced dose group (Low-dose octanoic acid supplement) was established to test whether a similar effect would be observed at a lower dose. Among the MCFAs, octanoic acid and decanoic acid have been reported to have different effects on mitochondrial function (17, 24). High-dose octanoic acid supplement comprised MCTs rich in octanoic acid. Therefore, MCTs rich in decanoic acid (Decanoic acid supplement) were used to examine differences in the effects on subjective health between the consumption of MCTs rich in decanoic acid and MCTs rich in octanoic acid. LCTs in edible oils consumed daily were used as control.

### 2.7 Physical exercise intervention

During the study period, participants were instructed to walk for 40 ± 10 min on two days each week and to record their performance on the lifestyle questionnaire. They were instructed to maintain their habitual walking speed. Exercise was to be performed on non-consecutive days of the week whenever possible. For safety reasons, the following indoor exercises were used as an alternative on days when it was raining or extremely hot.

1. Subjects having access to a walking machine, treadmill, etc., could use it to walk at a normal walking speed for the prescribed duration.
2. For subjects having no such facilities, foot stamping on a flat surface for a specified duration at a pace equivalent to habitual walking on the spot, without moving, was an acceptable alternative to walking outdoors.
3. If such alternative indoor exercise was carried out, it was to be recorded on the lifestyle questionnaire.

If any exercise could not be carried out for some unavoidable reason, the reason was to be recorded on the lifestyle questionnaire.

In a Japanese guideline (the Physical Activity Reference for Health Promotion 2013), normal walking was introduced as 3.0 metabolic equivalents (METs), strolling as 3.5 METs, and walking at a slightly faster pace as 4.3 METs (25). In this study, neither slow nor fast walking was instructed, and walking at a habitual pace was encouraged. Therefore, the subjects’ exercise was estimated to be 3.0–3.5 METs, which was defined as moderate intensity walking.

### 2.8 Lifestyle survey

Participants were asked to report their living conditions during the study period, such as consumption of the test diet, physical condition, medication, exercise, and alcohol consumption, using the lifestyle questionnaire.

### 2.9 Adverse events

During the study period, adverse events were investigated daily via the lifestyle questionnaire. If an adverse event occurred, the investigator immediately took the necessary and appropriate action, assessed the adverse event, and graded it for causal relation to the test diets as follows: none; probably none; may have; probably yes; yes; not assessable. Events other than those judged by the investigator to be “probably none” or “none” in causal relation to the test diets were considered to be adverse events.

### 2.10 Dietary survey

During the 12-week intervention period, dietary surveys of the normal diet without supplements were conducted before the intervention, and for four, eight, and 12 weeks after the intervention. Dietary surveys were conducted using a brief, self-administered diet history questionnaire (BDHQ).

### 2.11 Measurements

During the 12-week intervention period, subjective health and knee extension strength were measured before the intervention, and four weeks, eight weeks, and 12 weeks after the intervention.

The SF-36v2 (Japanese standard version), a HRQOL questionnaire, was used to assess subjective health. The SF-36 is the world’s most widely used questionnaire to assess subjective health, and its reliability and validity have been scientifically verified in Japan. It measures subjective health in eight subscales: physical function (PF), role physical (RP), bodily pain (BP), general health perception (GH), vitality (VT), social functioning (SF), role emotional (RE), and mental health (MH). In addition, the eight subscales can be analyzed together into three component summary scores: the physical component summary (PCS) consisting of PF, BP, GH, RP, SF, and VT; mental component summary (MCS) consisting of BP, GH, SF, RE, VT, and MH; and role-social component summary (RCS) consisting of BP, GH, RP, SF, and RE (26, 27).

Knee extension strength of both right and left legs was measured, using a lower limb muscle strength measuring device (Locomo Scan-II; ALCARE Co., Ltd., Tokyo, Japan).

### 2.12 Statistical analysis

The analysis was performed in accordance with the statistical analysis plan.

Subject characteristics and nutrient intakes calculated from BDHQs are shown as mean ± standard deviation. For HRQOL survey score data, actual value and change values, which are post-intervention values (at four, eight, and 12 weeks of intake) relative to before the intervention (week 0 of intake), are shown as mean ± standard deviation.

Multiple comparisons were made of nutrient intakes during the intervention period, to compare the control supplement and MCTs supplements. First, the equal variance was tested for each data set, using Levene’s test. If Levene’s test result was significant, Steel’s test was conducted; if not significant, Dunnett’s test was conducted.

HRQOL survey score data were first compared among the four groups by multiple comparisons for the values at week 0.

If there were no significant differences, a linear mixed model was used, with diet and time as fixed effects and subject as a random effect, to test for interactions between diet and time effects for the longitudinal measurements (at four, eight, and 12 weeks of intake). If no interaction was found, a linear mixed model equation without an interaction term was used to test for the diet effect for the longitudinal measurements. If a diet effect was found, multiple comparisons were performed between the four groups at each intervention period (four, eight, and 12 weeks of intake). If an interaction was found, a one-way analysis of variance was used to confirm the dietary effect. If there were significant differences, multiple comparisons were performed between the four groups at each intervention period (four, eight, and 12 weeks of intake). Multiple comparisons between the four groups were first tested for equal variance by Levene’s test. We then performed Steel’s test if equivariance was found, or Dunnett’s test if no equivariance was found.

For within-group comparisons of data at weeks 4, 8, and 12, based on week 0, equal variance was tested by Levene’s test, and multiple comparisons by Steel’s test if significant, or by Dunnett’s test if not.

Descriptive statistics of the analyzed data were calculated using Microsoft Excel for Office 365 MSO (Microsoft Japan Corporation, Tokyo, Japan). R statistical software, v4.1.0 for Windows (R Core Team, Vienna, Austria) was used for statistical processing. In all significant difference analyses, a risk ratio of less than 5% was considered significant, and a risk ratio of 5–10% was considered a tendency.

### 2.13 Exploratory analysis

A Pearson’s correlation test was conducted to determine the relationship between subjective health and muscle strength for exploratory analysis. Correlation coefficients were calculated using change values from 0 to 12 weeks in the SF-36v2 score and knee extension strength data as variables. In addition, we calculated partial correlation coefficients for SF-36v2 and knee extension strength, adjusting for gender, age, and fat free mass as covariates. Regarding gender, we assigned dummy variables as male = 1 and female = 2 for the analysis.

## 3 Results

Some of the data from this study (nutrients intake and knee extension strength) has been reported previously (21).

### 3.1 Analysis of subjects

**Figure 1:**
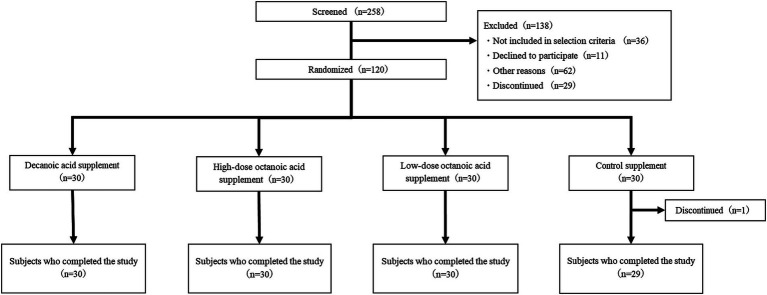
Flowchart of study subjects.

A flowchart of the subjects of the analysis is shown in Figure 1. Those who gave written consent (*n* = 258) were assessed for eligibility and 138 were excluded. The eligible subjects (*n* = 120) were enrolled and randomized into four groups of 30. Of those who completed the study (*n* = 119), those excluded by the statistical analysis plan were included in the analysis as per protocol set (PPS) (*n* = 112). A characteristic of the study participants has been described previously (21).

In this study, compliance was properly maintained. As a result, 89.9% of the subjects consumed 2 packets of test supplement daily during intervention. The remaining subjects also had an intake rate of over 98%. In addition, during the study period, walking was performed by all participants two days per week, although some participants performed the alternative indoor exercise due to the weather.

### 3.2 Adverse events

There were no adverse events attributed to the test supplement during this study.

### 3.3 Nutrient intake

There were no significant differences in nutrient intakes between the control supplement group and MCTs supplement groups, before or during the study period. Nutrient intakes were reported previously (21).

### 3.4 Test results of SF-36v2

The actual values and change values in each SF-36v2 score are shown in Table 2. There were no significant differences in any of the pre-intervention scores between the control group and MCTs supplement groups. In the control group there was no significant change in any of the SF-36 scores during the intervention period, compared to the pre-intervention period. As a result of the 12-week intervention, physical function (PF), general health (GH), vitality (VT) and mental component summary (MCS) showed significant increases in scores in all MCT supplement groups compared to the control group, either in actual or change scores. In addition, there was a significant increase in mental health (MH) scores in the Decanoic acid supplement and Low-dose octanoic acid supplement groups, compared to the control group. There were no significant changes in role-physical (RP), body pain (BP), social functioning (SF) and role-mental (RE), nor in physical component summary (PCS) or role-social component summary (RCS).

**Table 2:** Variation in SF-36v2 score with consumption of MCT supplements or control supplement.^1^

	Week	Control supplement		Decanoic acid supplement		High-dose octanoic acid supplement		Low-dose octanoic acid supplement	
		Actual value	Change value	Actual value	Change value	Actual value	Change value	Actual value	Change value
Physical functioning (PF)	0	90.7 ± 1.7		90.7 ± 1.7		93.0 ± 1.6		95.0 ± 0.9	
	4	89.5 ± 1.8	−1.2 ± 0.8	94.1 ± 1.3 *	3.3 ± 1.6	95.4 ± 1.2 *	2.3 ± 1.0	96.8 ± 0.7 *	1.8 ± 0.6
	8	91.9 ± 1.9	1.2 ± 1.4	94.4 ± 1.3	3.7 ± 1.2	96.6 ± 0.9 *	3.6 ± 1.1	97.0 ± 0.6 *	2 ± 0.8
	12	92.2 ± 1.5	1.6 ± 1.1	96.1 ± 1.1 *	5.4 ± 1.6	96.8 ± 1.0 *	3.8 ± 1.0	97.9 ± 0.5 *	2.9 ± 0.7
Role-physical (RP)	0	91.8 ± 2.6		96.3 ± 2.1		92.6 ± 3.2		95.8 ± 2.0	
	4	95.3 ± 1.7	3.5 ± 1.6	95.8 ± 2.3	−0.5 ± 1.4	97.3 ± 1.6	4.7 ± 2.7	97.1 ± 1.5	1.3 ± 1.5
	8	95.1 ± 1.9	3.2 ± 2.1	96.3 ± 2.1	0.0 ± 1.2	97.5 ± 1.2	4.9 ± 2.6	98.4 ± 1.3	2.7 ± 1.6
	12	95.0 ± 1.9	3.2 ± 1.7	97.5 ± 1.3	1.2 ± 1.1	98.9 ± 0.7	6.2 ± 2.7	98.9 ± 0.7	3.1 ± 1.7
Bodily pain (BP)	0	82.6 ± 3.0		83.1 ± 3.3		83.4 ± 2.8		84.5 ± 3.5	
	4	85.2 ± 3.0	2.6 ± 2.8	86.6 ± 3.1	3.4 ± 3.3	87.7 ± 2.4	4.4 ± 2.1	92.0 ± 2.2	7.5 ± 2.7
	8	86.3 ± 2.9	3.7 ± 2.1	92.4 ± 2.0	9.3 ± 3.2	88.9 ± 2.5	5.5 ± 2.4	91.6 ± 2.9	7.2 ± 2.5
	12	88.7 ± 2.9	6.1 ± 2.5	93.6 ± 2.1	10.5 ± 3.5	90.6 ± 2.3	7.3 ± 1.8	93.9 ± 2.4	9.4 ± 2.6
General health (GH)	0	73.4 ± 2.0		78.9 ± 2.4		78.7 ± 2.5		80.0 ± 2.5	
	4	72.9 ± 2.4	−0.5 ± 1.3	80.8 ± 2.6	2.0 ± 1.4	81.4 ± 2.6 *	2.7 ± 1.3	81.8 ± 2.5 *	1.8 ± 0.8
	8	73.7 ± 2.4	0.3 ± 1.4	83.1 ± 2.5*	4.3 ± 1	82.0 ± 2.7	3.3 ± 1.7	83.5 ± 3.0 *	3.6 ± 1.5
	12	72.9 ± 2.4	−0.5 ± 1.4	84.0 ± 2.3 *	5.1 ± 1.2 *	83.6 ± 2.7 *	4.9 ± 1.9	85.6 ± 2.5 *	5.6 ± 1.3 *
Vitality (VT)	0	74.0 ± 2.8		74.1 ± 2.6		75.0 ± 2.2		78.2 ± 2.3	
	4	75.9 ± 3.1	1.9 ± 1.6	79.2 ± 3.1	5.1 ± 1.5	80.4 ± 2.0	5.4 ± 1.6	82.2 ± 2.2	4.0 ± 1.3
	8	74.2 ± 3.6	0.2 ± 2.1	79.2 ± 2.5	5.1 ± 0.9	81.1 ± 2.3	6.0 ± 1.8 *	84.2 ± 2.2 *	6.0 ± 1.4 *
	12	73.7 ± 3.3	−0.2 ± 2.2	82.9 ± 2.3 *	8.8 ± 1.3 *	81.9 ± 2.3	6.9 ± 1.9 *	86.4 ± 2.2 *	8.2 ± 1.7 *
Social functioning (SF)	0	92.2 ± 2.9		88.0 ± 3.9		91.1 ± 3.1		94.2 ± 2.4	
	4	93.5 ± 2.5	1.3 ± 2.1	94.4 ± 3.0	6.5 ± 2.8	98.7 ± 1.0	7.6 ± 2.6	97.8 ± 1.6	3.6 ± 2.1
	8	95.3 ± 2.0	3.0 ± 1.9	98.1 ± 1.1	10.2 ± 3.6	98.7 ± 1.0	7.6 ± 2.6	98.7 ± 1.0	4.5 ± 2.4
	12	95.3 ± 2.4	3.0 ± 1.8	97.7 ± 1.2	9.7 ± 3.9	98.7 ± 1.0	7.6 ± 2.6	100.0 ± 0.0	5.8 ± 2.4
Role-emotional (RE)	0	93.4 ± 2.0		93.2 ± 3.3		93.5 ± 2.2		95.8 ± 1.7	
	4	94.3 ± 1.9	0.9 ± 1.3	96.0 ± 2.3	2.8 ± 3.6	96.7 ± 1.5	3.3 ± 1.7	99.4 ± 0.6	3.6 ± 1.6
	8	93.7 ± 2.4	0.3 ± 2.2	98.1 ± 1.3	4.9 ± 3.0	96.1 ± 1.7	2.7 ± 1.6	99.4 ± 0.4	3.6 ± 1.7
	12	95.4 ± 1.8	2.0 ± 1.6	97.2 ± 1.5	4.0 ± 2.4	97.0 ± 1.4	3.6 ± 1.7	100.0 ± 0.0	4.2 ± 1.7
Mental health (MH)	0	82.1 ± 2.4		84.8 ± 2.0		83.4 ± 1.9		84.5 ± 2.4	
	4	83.4 ± 2.3	1.4 ± 1.4	86.3 ± 2.2	1.5 ± 1.4	86.8 ± 1.7	3.4 ± 1.6	88.9 ± 1.8	4.5 ± 1.5
	8	84.3 ± 2.2	2.2 ± 1.9	89.6 ± 1.2	4.8 ± 1.3	87.1 ± 1.8	3.8 ± 1.2	90.2 ± 1.6 *	5.7 ± 1.8
	12	83.3 ± 2.0	1.2 ± 1.8	91.5 ± 1.0*	6.7 ± 1.6	89.1 ± 1.9	5.7 ± 1.3	91.6 ± 1.8 *	7.1 ± 2.0
Physical component summery (PCS)	0	50.8 ± 1.1		52.6 ± 1.1		52.8 ± 1.0		53.4 ± 1.0	
	4	50.6 ± 1.1	−0.3 ± 0.6	53.2 ± 0.9	0.6 ± 1.2	53.4 ± 0.8	0.6 ± 0.6	54.2 ± 0.7 *	0.8 ± 0.8
	8	51.5 ± 0.9	0.6 ± 0.7	53.6 ± 0.8	1.0 ± 0.9	54.1 ± 0.8	1.3 ± 0.7	54.3 ± 0.9	0.8 ± 0.9
	12	52.0 ± 1.0	1.1 ± 0.8	54.4 ± 0.8	1.8 ± 0.9	54.5 ± 0.8	1.7 ± 0.7	54.9 ± 0.7 *	1.4 ± 0.8
Mental component summery (MCS)	0	59.1 ± 1.1		60.0 ± 1.2		60.2 ± 1.0		60.8 ± 1.2	
	4	59.6 ± 1.3	0.5 ± 0.7	61.8 ± 1.3	1.7 ± 0.7	62.1 ± 1.0	1.9 ± 0.6	63.0 ± 1.2	2.2 ± 0.5
	8	59.5 ± 1.3	0.4 ± 0.9	63.2 ± 0.9	3.2 ± 0.6 *	62.4 ± 1.1	2.2 ± 0.6	63.8 ± 1.3 *	3.1 ± 0.8 *
	12	59.0 ± 1.3	−0.1 ± 0.9	64.4 ± 0.9 *	4.4 ± 0.7 *	63.2 ± 1.2	3.0 ± 0.6 *	65.0 ± 1.3 *	4.3 ± 0.8 *
Role-social component summery (RCS)	0	51.1 ± 1.1		49.9 ± 1.5		49.7 ± 1.3		50.9 ± 1.0	
	4	52.2 ± 1.1	1.0 ± 0.6	50.7 ± 1.2	0.9 ± 1.1	51.8 ± 0.7	2.1 ± 1.1	51.6 ± 0.8	0.7 ± 0.8
	8	52.1 ± 1.0	0.9 ± 0.9	51.3 ± 0.9	1.4 ± 1.1	51.3 ± 0.7	1.6 ± 1.1	51.7 ± 0.9	0.8 ± 0.8
	12	52.4 ± 1.0	1.3 ± 0.7	50.6 ± 0.7	0.7 ± 1.1	51.3 ± 0.7	1.7 ± 1.1	51.4 ± 0.7	0.5 ± 0.9

^1^Values are shown as mean ± standard deviation. *Significant difference compared to control group (p < 0.05, Dunnett test).

### 3.5 Correlation between SF-36v2 score and knee extension strength

The details of knee extension strength were reported previously (21). The results of the knee extension muscle strength measurements at 0 and 12 weeks are shown in Table 3. At week 12 of the intervention, all MCT supplement groups showed an increase from baseline in right knee extension strength, compared to the control group. For left knee extension strength, only the Decanoic acid supplement and Low-dose octanoic acid supplement groups showed higher values than the control group. The correlation coefficients between knee extension strength and SF-36 are shown in Table 4. A weak positive correlation was found between knee extension strength and MCS in Decanoic acid supplement group (0.40, *p* < 0.05). There was a positive correlation between knee extension strength and VT in Low-dose octanoic acid supplement group (0.42, *p* < 0.05). In contrast, in High-dose octanoic acid supplement group, a small positive correlation was found between right knee extension strength and PF (0.38, *p* < 0.05), but no correlation was found between VT, MH, MCS, and knee extension strength. This trend was similar when partial correlations were calculated using age, sex, and fat free mass as adjustment variables (Supplementary Tables S1–S4).

**Table 3:** Muscle strength of each group during the intervention period and their changes from the baseline.^1^

		Control supplement		Decanoic acid supplement		High-dose octanoic acid supplement		Low-dose octanoic acid supplement	
	Week	Actual values	Change values	Actual values	Change values	Actual values	Change values	Actual values	Change values
Knee extension strength									
Right, *N*	0	309.3 ± 21.0		306.4 ± 19.6		326.3 ± 30.1		254.6 ± 19.9	
	12	356.7 ± 16.5	47.3 ± 14.7	426.0 ± 20.3^†^	119.6 ± 17.3	456.8 ± 26.3^*,†^	130.5 ± 18.3	391.3 ± 23.0^†^	136.7 ± 19.1
Left, *N*	0	291.8 ± 19.9		308.0 ± 21.1		323.0 ± 24.3		244.0 ± 18.1	
	12	356.6 ± 20.1	64.8 ± 15.9	433.0 ± 20.2^*,†^	125.1 ± 13.0	441.6 ± 23.1^*,†^	118.6 ± 17.9	379.6 ± 20.7^†^	135.6 ± 21.7

^1^Values are shown as mean ± standard deviation.

^*^Significantly different from control group (*p* < 0.05, Dunnett test).

^†^Significantly different from the baseline (*p* < 0.05, Dunnett test).

**Table 4:** Correlation coefficients between knee extensor strength and SF-36 score at 12 weeks of intervention.

	Control supplement		Decanoic acid supplement		High-dose octanoic acid supplement		Low-dose octanoic acid supplement	
	Right knee extension	Left knee extension	Right knee extension	Left knee extension	Right knee extension	Right knee extension	Right knee extension	Left knee extension
Physical functioning (PF)	0.03	−0.10	−0.05	−0.01	0.22	0.38 *	0.36	0.25
Role-physical (RP)	0.23	0.06	−0.03	−0.10	−0.13	−0.02	0.25	0.23
Bodily pain (BP)	0.33	−0.18	0.53 **	0.04	−0.05	−0.14	−0.03	0.21
General health (GH)	0.34	0.18	0.39 *	0.57 **	0.12	0.03	0.16	0.11
Vitality (VT)	0.25	0.05	0.14	−0.06	−0.01	−0.04	0.42 *	−0.03
Social functioning (SF)	−0.08	0.22	0.27	−0.09	0.16	0.20	0.30	0.17
Role-emotional (RE)	0.13	0.14	0.21	0.34	−0.01	0.08	0.10	−0.23
Mental health (MH)	0.03	0.10	0.06	−0.08	−0.08	0.04	0.15	−0.17
Physical component summery (PCS)	0.33	−0.21	0.17	0.16	0.05	0.04	−0.02	0.33
Mental component summery (MCS)	0.23	0.23	0.40*	0.40 *	0.06	0.06	0.24	0.24
Role-social component summary (RCS)	−0.17	0.25	0.07	−0.02	−0.04	0.11	0.23	−0.07

*p < 0.05 (Pearson’s correlation coefficient). **p < 0.01 (Pearson’s correlation coefficient).

## 4 Discussion

In this study, the enrolled subjects were aged 60–74 years, had no exercise habits, and had an average BMI of 21.5 kg/m^2^, which is generally below average for Japanese people of that age group (28). The intervention consisted of continuous supplements of MCTs combined with moderate-intensity equivalent walking for 12 weeks. The results showed significant improvements in scores on several subscales of the SF-36 in all MCTs supplement groups, compared to the control group. Therefore, regardless of the octanoic and decanoic acid composition, it is speculated that the combination of a continuous daily intake of 2 g or more of MCTs and walking may have an effect on subjective health and HRQOL in healthy middle-aged and older adults with lower BMIs and no exercise habits.

This study evaluated the effects of continuous MCTs intake and a moderate-intensity exercise intervention on mental and physical aspects of subjective health and social functioning, using the SF-36. The scoring of the subjects, based on the national standard score of the SF-36 at baseline (26), showed that all scores were above 50 points. There were significant increases in PF, GH, VT, and MCS in all MCTs supplement groups at week 12, compared to the control. MH also increased significantly in Decanoic acid supplement and Low-dose octanoic acid supplement groups. On the other hand, there were no significant changes in RP or BP, and there was no change in PCS, a summary score of physical health. The results indicated that the biggest effects of this intervention were in improving the mental aspect of QOL among the mental, physical, and social aspects of health. It also suggested improvements in PF, GH, and VT, which are subscales of PCS, and in GH, which is a subscale of RCS. Therefore, MCTs intake and walking have been shown to potentially improve mental aspects of QOL in middle-aged and older adults with no exercise habits and low BMIs.

Previous studies have shown a correlation between physical activity and HRQOL scores (29, 30). A study of healthy individuals aged 60–89 years in the U.S. reported higher HRQOL scores in the group that engaged in more than one hour of moderate-intensity physical activity per week, compared with the group that engaged in less than one hour per week (30). Referring to previous studies in the U.S., the present study predicted an improvement in HRQOL scores with increased physical activity. However, our control group showed no significant changes in all SF-36 items, as measured before and after the intervention.

On the other hand, because middle-aged and older adults with no exercise habits were subjected to exercise, there was a possibility that mental and physical fatigue associated with the walking exercise might affect the SF-36 score. However, the control group in this study showed no significant changes in physical health, mental status, or items representing the frequency of feeling tired. It is possible that in addition to the increase in physical activity, the consumption of MCTs may have affected subjective health.

In Japan it is recommended to exercise at least two days a week, for at least 30 min per session, to reduce the risk of age-related decline in functional capability in daily living, and to extend the period of independent living by increasing the intensity and frequency of physical activity (31). This is comparable to the exercise load in the present study, but no participants dropped out of the study because of the walking exercise intervention. Therefore, the results may be valuable in that continued MCTs intake, combined with relatively easy exercise, may have improved HRQOL in the subjects of this study.

There were significant increases in the MCTs supplement groups, compared to the control, especially in the subscales of mental aspects of health, such as VT, MH and GH. Their summary score, MCS, was also significantly increased. In a study investigating the effects of MCTs on mental health, the effects of medium-and long-chain triglycerides (MLCTs) on depression-like symptoms were reported in animal studies. One study suggested that the intake of MLCTs alleviated depression-like behavior and had an antidepressant-like effect compared to the intake of LCTs in mice subjected to the stress of continuous forced swimming (32). This trend is similar to the result of the present study, which shows a significant improvement in MH and MCS scores, which indicate a depressed mental state. It is speculated that consumption of MCTs may affect subjective health, especially the mental aspect of health.

On the other hand, there was a significant change in PF, a subscale that significantly affects the physical aspect of health, although there was no change in PCS. The PF-related questions include an assessment of the walking and score has been reported to correlate with walking function (33). Therefore, in addition to the improvement in walking function resulting from the walking exercise as an intervention, lowering the psychological barrier to walking is thought to be one of the reasons for the increased PF score. Additionally, in the MCTs supplement groups, there was a significant increase in lower limb strength after the intervention, compared to the control group. It has been reported that continuous intake of MCTs increases metabolism-related enzymes in skeletal muscle (14, 34). Therefore, in our MCTs supplement groups, it is hypothesized that increased fatty acid oxidation capacity provided an ample energy supply for muscle activation in skeletal muscle. This increase in energy supply may have contributed to the improvement in muscle strength, consequently leading to the increase in PF scores.

The SF-36 is an assessment that measures subjective physical and mental health, but it is also used to assess subjective fatigue. Subjective fatigue is a subjective feeling of awareness of the presence of physical and mental fatigue. VT, which represents the frequency of experiencing fatigue, has been used as a measure of physical fatigue in several clinical studies and its validity has been reported (35, 36). In addition, MH is an indicator of levels of anxiety and depression, which have been reported to correlate with fatigue (37, 38). Moreover, both MH and MCS have been reported to have a strong negative correlation with mental fatigue, a subscale of the MFI-20 used as a fatigue assessment method (MCS *r* = −0.563, MH -0.550, VT -0.574, SF -0.500) (39). Intake of coenzyme Q10, which has been reported to have fatigue-reducing effects, resulted in increased SF-36 VT, MH, and MCS scores, alongside a decreased salivary secretory immunoglobulin A secretion rate — a biological indicator of stress closely related to fatigue (40). In addition, VT has been used as a measure of physical fatigue, while MH and MCS have been used as indicators of mental fatigue (41). Although biomarkers were not evaluated in the present study, and the direct relationship to fatigue is not clear, significant increases in VT, MH, and MCS were observed in the MCTs supplement groups. It can be speculated that the combination of MCTs intake and moderate-intensity exercise may reduce subjective physical and mental fatigue in middle-aged and older adults. It is also considered that this reduction in fatigue might contribute to the improvements in SF-36 scores.

Several mechanisms have been proposed for the perception of fatigue. It has been suggested that decreased adenosine triphosphate (ATP) production due to mitochondrial dysfunction, and cellular inflammation caused by oxidative stress, are related to fatigue (42, 43). Coenzyme Q10 and imidazole dipeptide are food ingredients that reduce mental and physical fatigue. These mechanisms of action have been reported to increase in mitochondrial ATP production and antioxidant effects (44), as well as anti-inflammatory effects via antioxidant effects (45).

Excessive production of reactive oxygen radicals in muscle and nerve cells due to over-exertion causes oxidation and damage to cells and intracellular proteins and lipids. Whether it is exercise-induced fatigue or psychological work-related fatigue, cytokines produced by the immune system cells that detect this damage are sent to the cranial nervous and endocrine systems, leading to the perception of fatigue. It has been suggested that fatigue can be delayed if sufficient energy is not available to repair the cells at that time (46). Indeed, increased levels of oxidative stress and decreased efficiency of mitochondrial ATP production have been reported in patients with chronic fatigue syndrome (CFS) (47–49). Providing sufficient ATP by maintaining mitochondrial function and suppressing oxidative stress *in vivo* may be important in reducing subjective fatigue, as it leads to a reduction in fatigue.

Studies have reported that MCTs or octanoic acid and decanoic acid each work to increase mitochondrial biosynthesis and activation of metabolism-related enzymes in tissues such as the cranial nervous system and skeletal muscle (14, 17, 24, 34, 50) and decrease oxidative stress (46). Moreover, β-hydroxybutyrate, a ketone converted from MCT, has been reported to increase protein expression of Mn-SOD and catalase (51). Therefore, we speculate that such an increase in ATP production and antioxidant capacity may have been the underlying mechanisms for the improvement in SF-36 scores in this study, indicating alleviation of subjective fatigue. However, further research is needed to elucidate these mechanisms.

In this study we examined the effects of MCT dose and fatty acid composition on HRQOL, in combination with walking exercise. Our Low-dose octanoic acid supplement group, which had the lowest daily MCTs intake, and Decanoic acid supplement group, which had an octanoic acid intake similar to the Low-dose octanoic acid supplement group, had significant changes in several SF-36 items, compared to the control group. The High-dose octanoic acid supplement group had fewer items that showed significant differences from the control. One reason for this may have been differences in muscle mass and strength at baseline. Although no statistically significant differences were found between the groups, knee extension strength at week 0 was approximately 20 N and 70 N greater in the High-dose octanoic acid supplement group than in the Decanoic acid supplement and High-dose octanoic acid supplement groups, respectively (Table 3). In the Decanoic acid supplement and Low-dose octanoic acid supplement groups there were significant increases in both left and right knee extension strengths at the change value, compared to the control diet group. No significant change in right knee extension strength was observed in the High-dose octanoic acid supplement group. The high muscle mass at baseline may have had at least a small effect on this result.

We analyzed the correlations between the changes in knee extension strength and SF-36 scores during the intervention. We found a weak to moderate positive correlation in scores related to subjective fatigue (such as MCS or VT) only in the Decanoic acid supplement and Low-dose octanoic acid supplement groups. This means there was an association between increased lower limb muscle strength and improvement in the mental subjective health subscale in the Decanoic acid supplement and Low-dose octanoic acid supplement groups. It is speculated that the increase in lower limb muscle strength associated with MCTs consumption may have had a small positive effect on subjective health and fatigue. It has been reported that SF-36 scores are more likely to decrease in individuals with reduced muscle strength (52), suggesting that physical function correlates with HRQOL. In conclusion, these results suggest that a certain daily intake (1.24 g) of octanoic acid as MCFA, which constitutes MCTs, in combination with walking exercise, might improve subjective health and reduce subjective fatigue by increasing muscle strength in middle-aged and older adults with low BMIs and no exercise habits.

Octanoic acid has been shown to increase the amount of acylated ghrelin in the stomach (53), and to activate Akt/mTOR, which is involved in protein metabolism (15). In fact, it has been reported that continuous intake of octanoic acid-rich MCTs increased plasma albumin concentrations (11), skeletal muscle mass and strength (12, 13). Therefore, it has been speculated that the antioxidant effect of albumin (54) and the increase in muscle strength may have influenced the present results. On the other hand, the mechanism by which decanoic acid intake increases muscle strength is not fully understood. This study was also unable to clarify the effects of different amounts of decanoic acid intake on HRQOL and subjective fatigue. Further studies are required, to investigate the mechanism by which decanoic acid affects muscle strength in animals and humans, to confirm the detailed dose–response. In this case, to establish the effects of decanoic acid dose, muscle strength and muscle mass at the baseline should be considered when assigning subjects.

Other factors that may affect this study include the fact that MCTs have been reported to be more likely to cause gastrointestinal discomfort than LCTs (55). However, the discomfort of ingesting MCTs was not considered to have a significant impact because the subjects’ adherence to supplement consumption during the study period was high (over 90%) and they did not report any discomfort.

In this study, healthy men and women with no exercise habits undertook walking, a moderate-intensity exercise, twice a week. SF-36 was evaluated before and after the intervention. There are several limitations to the study. Firstly, whether walking or MCT had a greater effect on HRQOL is not clear. Secondly, the effect on middle-aged and older adults with a habit of daily exercise is unknown. Thirdly, the effect on subjective health at moderate or higher exercise intensities is unknown. Lastly, the effect on fatigue is unknown because biomarkers of fatigue were not measured.

## 5 Conclusion

Using SF-36v2, this study investigated the effects of 12 weeks of MCTs supplements plus walking equivalent to moderate-intensity exercise twice a week on subjective health and HRQOL in middle-aged and older adults with no exercise habits. The results showed that regardless of the MCFA composition of the MCTs and the amount of MCTs, the combination of continuous intake of 2 g or more of MCTs per day and walking exercise significantly improved scores on subscales of the physical aspect of QOL and summary scores on the mental aspect of QOL, compared with the LCTs intake used as a comparison. It is estimated that continuous intake of MCTs and walking might have affected HRQOL and improved physical and mental subjective health in middle-aged and older healthy adults with low BMIs.

## Data availability statement

The original contributions presented in the study are included in the article/Supplementary material, further inquiries can be directed to the corresponding author.

## Ethics statement

The studies involving humans were approved by Yoga Allergy Clinic (approval number 21000023, approval date May 13, 2022). The studies were conducted in accordance with the local legislation and institutional requirements. The participants provided their written informed consent to participate in this study.

## Author contributions

HI: Conceptualization, Formal analysis, Writing – original draft. KK: Conceptualization, Investigation, Writing – review & editing. SW: Project administration, Supervision, Writing – review & editing. NN: Conceptualization, Methodology, Supervision, Writing – review & editing. TM: Supervision, Writing – review & editing.

## Footnotes

^1^ https://rctportal.niph.go.jp/s/detail/um?trial_id=UMIN000046861
