# Supplementary material for: Effect of medium-chain triglycerides supplements and walking on health-related quality of life in sedentary, healthy middle-aged, and older adults with low BMIs: a randomized, double-blind, placebo-controlled, parallel-group trial
Source: Front Nutr. 2023 Nov 23;10:1296896. doi: 10.3389/fnut.2023.1296896 (PMC10701736; doi:10.3389/fnut.2023.1296896)
Supplement: Supplementary file 1 [file Table_1.docx]

Supplementary Material

Effect of medium-chain triglycerides supplements and walking on health-related quality of life in sedentary, healthy middle-aged and older adults with low BMIs: A randomized, double-blind, placebo-controlled, parallel-group trial

Haruna Ishikawa^*^, Keiichi Kojima, Shinji Watanabe, Naohisa Nosaka, Tatsushi Mutoh

*** Correspondence:** Haruna Ishikawa: h-ishikawa@nisshin-oillio.com

# Supplementary Table

**Supplementary Table 1. Partial correlation coefficient between knee extension strength and SF-36 score at 12 weeks of intervention in Control supplement group.**

|  |  | Control supplement | | | | | | | |
| --- | --- | --- | --- | --- | --- | --- | --- | --- | --- |
|  |  | correlation | | Adjustment variable | | | | | |
|  |  |  |  | Gender | | Age | | Fat free mass | |
|  |  | P value | correlation coefficient | P value | partial　correlation coefficient | P value | partial correlation coefficient | P value | partial correlation coefficient |
| Right knee extension | Physical functioning（PF） | 0.90 | 0.03 | 0.86 | 0.04 | 0.89 | 0.03 | 0.87 | 0.03 |
|  | Role-physical（RP) | 0.23 | 0.23 | 0.18 | 0.26 | 0.18 | 0.26 | 0.24 | 0.23 |
|  | Bodily pain（BP) | 0.08 | 0.33 | 0.08 | 0.33 | 0.09 | 0.32 | 0.14 | 0.29 |
|  | General health（GH) | 0.07 | 0.34 | 0.09 | 0.33 | 0.07 | 0.35 | 0.12 | 0.30 |
|  | Vitality（VT) | 0.19 | 0.25 | 0.21 | 0.24 | 0.17 | 0.27 | 0.31 | 0.20 |
|  | Social functioning（SF) | 0.67 | -0.08 | 0.66 | -0.09 | 0.73 | -0.07 | 0.74 | -0.06 |
|  | Role-emotional（RE) | 0.51 | 0.13 | 0.55 | 0.12 | 0.36 | 0.18 | 0.68 | 0.08 |
|  | Mental health（MH） | 0.87 | 0.03 | 0.85 | 0.04 | 0.79 | 0.05 | 0.90 | 0.03 |
|  | Physical component summery（PCS) | 0.08 | 0.33 | 0.07 | 0.34 | 0.10 | 0.32 | 0.12 | 0.30 |
|  | Mental component summery（MCS) | 0.22 | 0.23 | 0.25 | 0.23 | 0.22 | 0.24 | 0.35 | 0.18 |
|  | Role-social component summary（RCS) | 0.37 | -0.17 | 0.39 | -0.17 | 0.46 | -0.14 | 0.46 | -0.14 |
| Right knee extension | Physical functioning（PF） | 0.61 | -0.10 | 0.72 | -0.07 | 0.62 | -0.10 | 0.57 | -0.11 |
|  | Role-physical（RP) | 0.76 | 0.06 | 0.50 | 0.13 | 0.73 | 0.07 | 0.73 | 0.07 |
|  | Bodily pain（BP) | 0.35 | -0.18 | 0.36 | -0.18 | 0.34 | -0.19 | 0.66 | -0.09 |
|  | General health（GH) | 0.35 | 0.18 | 0.45 | 0.15 | 0.35 | 0.18 | 0.16 | 0.27 |
|  | Vitality（VT) | 0.81 | 0.05 | 0.92 | 0.02 | 0.80 | 0.05 | 0.39 | 0.17 |
|  | Social functioning（SF) | 0.24 | 0.22 | 0.27 | 0.22 | 0.24 | 0.23 | 0.30 | 0.20 |
|  | Role-emotional（RE) | 0.46 | 0.14 | 0.59 | 0.11 | 0.40 | 0.17 | 0.22 | 0.24 |
|  | Mental health（MH） | 0.61 | 0.10 | 0.54 | 0.12 | 0.59 | 0.11 | 0.56 | 0.11 |
|  | Physical component summery（PCS) | 0.27 | -0.21 | 0.33 | -0.19 | 0.26 | -0.22 | 0.41 | -0.16 |
|  | Mental component summery（MCS) | 0.22 | 0.23 | 0.79 | 0.05 | 0.67 | 0.08 | 0.32 | 0.19 |
|  | Role-social component summary（RCS) | 0.20 | 0.25 | 0.16 | 0.27 | 0.13 | 0.29 | 0.29 | 0.21 |

**Supplementary Table 2. Partial correlation coefficient between knee extension strength and SF-36 score at 12 weeks of intervention in Decanoic acid supplement group.**

|  |  | Decanoic acid supplement | | | | | | | |
| --- | --- | --- | --- | --- | --- | --- | --- | --- | --- |
|  |  | correlation | | Adjustment variable | | | | | |
|  |  |  |  | Gender | | Age | | Fat free mass | |
|  |  | P value | correlation coefficient | P value | partial correlation coefficient | P value | partial correlation coefficient | P value | partial correlation coefficient |
| Right knee extension | Physical functioning（PF） | 0.82 | -0.05 | 0.74 | -0.07 | 0.79 | -0.06 | 0.84 | -0.04 |
|  | Role-physical（RP) | 0.87 | -0.03 | 0.88 | -0.03 | 0.90 | -0.03 | 0.90 | -0.03 |
|  | Bodily pain（BP) | 0.00 | 0.53 | 0.00 | 0.56 | 0.00 | 0.55 | 0.00 | 0.54 |
|  | General health（GH) | 0.05 | 0.39 | 0.05 | 0.39 | 0.05 | 0.40 | 0.05 | 0.39 |
|  | Vitality（VT) | 0.48 | 0.14 | 0.58 | 0.12 | 0.50 | 0.14 | 0.33 | 0.20 |
|  | Social functioning（SF) | 0.17 | 0.27 | 0.18 | 0.27 | 0.19 | 0.26 | 0.14 | 0.30 |
|  | Role-emotional（RE) | 0.30 | 0.21 | 0.32 | 0.20 | 0.32 | 0.20 | 0.27 | 0.22 |
|  | Mental health（MH） | 0.75 | 0.06 | 0.85 | 0.04 | 0.77 | 0.06 | 0.67 | 0.09 |
|  | Physical component summery（PCS) | 0.39 | 0.17 | 0.37 | 0.18 | 0.36 | 0.19 | 0.40 | 0.17 |
|  | Mental component summery（MCS) | 0.04 | 0.40 | 0.05 | 0.39 | 0.04 | 0.41 | 0.02 | 0.45 |
|  | Role-social component summary（RCS) | 0.71 | 0.07 | 0.74 | 0.07 | 0.76 | 0.06 | 0.64 | 0.09 |
| Right knee extension | Physical functioning（PF） | 0.94 | -0.01 | 0.98 | 0.00 | 0.93 | -0.02 | 0.94 | -0.02 |
|  | Role-physical（RP) | 0.61 | -0.10 | 0.61 | -0.10 | 0.62 | -0.10 | 0.58 | -0.11 |
|  | Bodily pain（BP) | 0.85 | 0.04 | 0.89 | 0.03 | 0.84 | 0.04 | 0.86 | 0.04 |
|  | General health（GH) | 0.00 | 0.57 | 0.00 | 0.57 | 0.00 | 0.57 | 0.00 | 0.58 |
|  | Vitality（VT) | 0.76 | -0.06 | 0.81 | -0.05 | 0.75 | -0.07 | 0.68 | -0.09 |
|  | Social functioning（SF) | 0.65 | -0.09 | 0.65 | -0.09 | 0.65 | -0.09 | 0.62 | -0.10 |
|  | Role-emotional（RE) | 0.08 | 0.34 | 0.08 | 0.35 | 0.09 | 0.34 | 0.07 | 0.36 |
|  | Mental health（MH） | 0.69 | -0.08 | 0.74 | -0.07 | 0.70 | -0.08 | 0.64 | -0.10 |
|  | Physical component summery（PCS) | 0.43 | 0.16 | 0.45 | 0.15 | 0.42 | 0.16 | 0.43 | 0.16 |
|  | Mental component summery（MCS) | 0.04 | 0.40 | 0.82 | 0.05 | 0.85 | 0.04 | 0.85 | 0.04 |
|  | Role-social component summary（RCS) | 0.90 | -0.02 | 0.92 | -0.02 | 0.89 | -0.03 | 0.88 | -0.03 |

**Supplementary Table3. Partial correlation coefficient between knee extension strength and SF-36 score at 12 weeks of intervention in High-dose octanoic acid supplement group.**

|  |  | **High-dose octanoic acid supplement** | | | | | | | |
| --- | --- | --- | --- | --- | --- | --- | --- | --- | --- |
|  |  | **correlation** | | **Adjustment variable** | | | | | |
|  |  |  |  | Gender | | Age | | Fat free mass | |
|  |  | P value | correlation coefficient | P value | partial correlation coefficient | P value | partial correlation coefficient | P value | partial correlation coefficient |
| Right knee extension | Physical functioning（PF） | 0.26 | 0.22 | 0.19 | 0.26 | 0.45 | 0.15 | 0.14 | 0.29 |
|  | Role-physical（RP) | 0.52 | -0.13 | 0.61 | -0.10 | 0.56 | -0.12 | 0.63 | -0.10 |
|  | Bodily pain（BP) | 0.80 | -0.05 | 0.79 | -0.05 | 0.95 | -0.01 | 0.87 | -0.03 |
|  | General health（GH) | 0.54 | 0.12 | 0.49 | 0.14 | 0.43 | 0.16 | 0.41 | 0.17 |
|  | Vitality（VT) | 0.97 | -0.01 | 0.86 | 0.04 | 0.91 | -0.02 | 0.87 | 0.03 |
|  | Social functioning（SF) | 0.40 | 0.16 | 0.29 | 0.21 | 0.67 | 0.09 | 0.33 | 0.20 |
|  | Role-emotional（RE) | 0.95 | -0.01 | 1.00 | 0.00 | 0.93 | 0.02 | 0.96 | 0.01 |
|  | Mental health（MH） | 0.69 | -0.08 | 0.81 | -0.05 | 0.71 | -0.08 | 0.83 | -0.04 |
|  | Physical component summery（PCS) | 0.80 | 0.05 | 0.81 | 0.05 | 0.67 | 0.09 | 0.65 | 0.09 |
|  | Mental component summery（MCS) | 0.75 | 0.06 | 0.63 | 0.10 | 0.73 | 0.07 | 0.61 | 0.10 |
|  | Role-social component summary（RCS) | 0.86 | -0.04 | 0.97 | -0.01 | 0.72 | -0.07 | 0.92 | -0.02 |
| Right knee extension | Physical functioning（PF） | 0.04 | 0.38 | 0.03 | 0.42 | 0.12 | 0.31 | 0.03 | 0.43 |
|  | Role-physical（RP) | 0.91 | -0.02 | 0.98 | -0.01 | 0.99 | 0.00 | 0.97 | -0.01 |
|  | Bodily pain（BP) | 0.49 | -0.14 | 0.49 | -0.14 | 0.66 | -0.09 | 0.53 | -0.13 |
|  | General health（GH) | 0.87 | 0.03 | 0.83 | 0.04 | 0.70 | 0.08 | 0.80 | 0.05 |
|  | Vitality（VT) | 0.83 | -0.04 | 0.94 | -0.01 | 0.74 | -0.07 | 0.91 | -0.02 |
|  | Social functioning（SF) | 0.32 | 0.20 | 0.24 | 0.23 | 0.67 | 0.09 | 0.29 | 0.21 |
|  | Role-emotional（RE) | 0.70 | 0.08 | 0.67 | 0.09 | 0.52 | 0.13 | 0.66 | 0.09 |
|  | Mental health（MH） | 0.85 | 0.04 | 0.76 | 0.06 | 0.81 | 0.05 | 0.78 | 0.06 |
|  | Physical component summery（PCS) | 0.86 | 0.04 | 0.86 | 0.03 | 0.65 | 0.09 | 0.78 | 0.06 |
|  | Mental component summery（MCS) | 0.75 | 0.06 | 0.82 | -0.05 | 0.75 | -0.07 | 0.81 | -0.05 |
|  | Role-social component summary（RCS) | 0.57 | 0.11 | 0.50 | 0.13 | 0.73 | 0.07 | 0.55 | 0.12 |

**Supplementary Table4. Partial correlation coefficient between knee extension strength and SF-36 score at 12 weeks of intervention in High-dose octanoic acid supplement group.**

|  |  | **High-dose octanoic acid supplement** | | | | | | | |
| --- | --- | --- | --- | --- | --- | --- | --- | --- | --- |
|  |  | **correlation** | | **Adjustment variable** | | | | | |
|  |  |  |  | Gender | | Age | | Fat free mass | |
|  |  | P value | correlation coefficient | P value | partial correlation coefficient | P value | partial correlation coefficient | P value | partial correlation coefficient |
| Right knee extension | Physical functioning（PF） | 0.06 | 0.36 | 0.06 | 0.36 | 0.05 | 0.38 | 0.10 | 0.32 |
|  | Role-physical（RP) | 0.19 | 0.25 | 0.22 | 0.24 | 0.17 | 0.27 | 0.26 | 0.23 |
|  | Bodily pain（BP) | 0.90 | -0.03 | 0.80 | 0.05 | 0.99 | 0.00 | 0.79 | -0.05 |
|  | General health（GH) | 0.40 | 0.16 | 0.31 | 0.20 | 0.45 | 0.15 | 0.38 | 0.17 |
|  | Vitality（VT) | 0.03 | 0.42 | 0.04 | 0.39 | 0.03 | 0.41 | 0.03 | 0.41 |
|  | Social functioning（SF) | 0.12 | 0.30 | 0.06 | 0.36 | 0.15 | 0.28 | 0.14 | 0.29 |
|  | Role-emotional（RE) | 0.62 | 0.10 | 0.95 | -0.01 | 0.69 | 0.08 | 0.70 | 0.08 |
|  | Mental health（MH） | 0.45 | 0.15 | 0.67 | 0.09 | 0.52 | 0.13 | 0.53 | 0.13 |
|  | Physical component summery（PCS) | 0.91 | -0.02 | 0.80 | 0.05 | 0.92 | 0.02 | 0.82 | -0.05 |
|  | Mental component summery（MCS) | 0.22 | 0.24 | 0.23 | 0.24 | 0.27 | 0.22 | 0.24 | 0.23 |
|  | Role-social component summary（RCS) | 0.23 | 0.23 | 0.38 | 0.17 | 0.28 | 0.21 | 0.28 | 0.22 |
| Right knee extension | Physical functioning（PF） | 0.20 | 0.25 | 0.21 | 0.25 | 0.23 | 0.24 | 0.23 | 0.24 |
|  | Role-physical（RP) | 0.24 | 0.23 | 0.25 | 0.23 | 0.28 | 0.22 | 0.27 | 0.22 |
|  | Bodily pain（BP) | 0.28 | 0.21 | 0.28 | 0.22 | 0.34 | 0.19 | 0.30 | 0.21 |
|  | General health（GH) | 0.56 | 0.11 | 0.57 | 0.11 | 0.49 | 0.14 | 0.56 | 0.12 |
|  | Vitality（VT) | 0.88 | -0.03 | 0.89 | -0.03 | 0.95 | -0.01 | 0.84 | -0.04 |
|  | Social functioning（SF) | 0.40 | 0.17 | 0.41 | 0.16 | 0.31 | 0.20 | 0.42 | 0.16 |
|  | Role-emotional（RE) | 0.23 | -0.23 | 0.22 | -0.24 | 0.28 | -0.21 | 0.22 | -0.24 |
|  | Mental health（MH） | 0.40 | -0.17 | 0.41 | -0.17 | 0.49 | -0.14 | 0.38 | -0.18 |
|  | Physical component summery（PCS) | 0.08 | 0.33 | 0.08 | 0.34 | 0.12 | 0.31 | 0.09 | 0.33 |
|  | Mental component summery（MCS) | 0.22 | 0.24 | 0.74 | -0.07 | 0.85 | -0.04 | 0.72 | -0.07 |
|  | Role-social component summary（RCS) | 0.74 | -0.07 | 0.75 | -0.06 | 0.85 | -0.04 | 0.71 | -0.07 |
